# Lectures on the Diseases of Women

**Published:** 1861-12

**Authors:** 


### Book Notices.

---

Lectures on the Diseases of Women. By Charles West, M. D., Fellow of the Royal College of Physicians; Examiner in Midwifery at the Royal College of Surgeons of England, etc., etc. Second American, from the Second London Edition. Philadelphia: Blanchard and Lea. 1861.

If Bellona has shown herself no fostering mother of the peaceful literature of Esculapius, she, at least, deserves credit that fewer mediocre, and even worse, volumes have been foisted upon an impecunious and unsuspecting profession in the twelve months just ended, than in many of their predecessors. If fewer books are printed, and fewer journals issued, those hardy few must, certainly, have some strong savor of value, that they should have survived the rough nurture of an atmosphere laden with villainous saltpetre, and aglow with the red wrath of war. And so we set it down as certain, that Barwell and Bumstead, and Lawson and Bedford, are names that will live in our libraries, when peace shall once more fold her white wings and settle down amid the prairies and the forests and the mountains the guns of Sumter scared her from. An earnest, manly tone pervades the literature of to-day, before which fine-spun theories, glittering generalities and specious sophisms wilt and wither, like a dahlia-stem at the first frost.

Dr. Chas. West will need no introduction, at our hands, to most of our readers,—at least, to those who have followed, with any degree of zeal, the specialty of our author. This volume embraces, in its 483 handsomely printed pages, the first part of the *Diseases of Women*, which appeared in Messrs. Blanchard and Lea's *Medical News and Library* for 1857–58, and the second part, promised, at that time, to "treat of all the remaining diseases of the female system." This latter consists of twelve additional lectures, commencing with diseases of the parts connected with the uterus, and including ovarian tumors and dropsy,—to which six lectures are devoted,—and closing with three lectures on diseases of the female bladder, of the urethra and vagina, and the external organs of generation.

Of the first part of the volume,—given at the time of the publication, our opinion is already on record; but we have glanced over,—all we have as yet had time for,—with renewed pleasure, the introductory lectures on the symptoms of diseases of women, the examination of the various classes of symptoms and the modes of examination. And as a better exponent of his clear, impartial, logical manner, and a certain charm of simplicity and directness most admirable, we add, at the risk of making this notice too lengthy for our limits, the following extracts:

"But just as disorder of the functions of other organs not seldom attends upon the physiological processes going on in the womb, so may it follow upon uterine irritation produced by disease; and a large proportion of the most obstinate forms of dyspepsia, and a still larger number of hysterical and nervous affections have been excited and are kept up by disease of the womb. In a great many of these cases, minute inquiry elicits evidence of functional disorder of the generative organs, as shown by disturbed menstruation, by leucorrhœal discharges, or by painful sensations, although none of these symptoms may have been so marked as to have engaged the patient's notice; or she may have regarded them as trivial accidents not worth mention when compared with the other, and to her feelings the more important causes of her sufferings.*

Need I guard myself against being misunderstood, against being supposed to say that, in the management of a woman who is dyspeptic, your attention is to be turned less to the state of her stomach than to that of her womb; or that if a woman suffer from neuralgia, you are at once to suspect the existence of uterine disease? I mean no such thing; but *what I do mean* is, that, in the treatment of diseases occurring among the patients of the female sex, you should always bear in mind that, besides the ordinary causes of disease common to both sexes, there is another set of causes peculiar to themselves. Whenever, therefore, the ordinary principles of pathology fail to explain, or the ordinary proceedings of therapeutics prove inadequate to cure the ailments of any female patient, it behooves you to remember that, in her sex, and in its peculiar diseases, you may perhaps find a clue to the cause of her present symptoms, and discover indications which may show you how to accomplish their cure."

The following paragraph closes the lecture on the modes of examination, and is so candid, so just, so moderate an estimate of that much used and abused instrument,—the speculum, as to, at once, redeem it from the odium its abusers subject it to. The quotation is, in part, his answer to the broad question, "What is your opinion of the speculum?"—

"In estimating the value of the speculum as a means of diagnosis, I think that the advances in knowledge of uterine disease, of which it was the indirect occasion by the impulse which it gave to their study, are sometimes confounded with

---

*In vol. ii. of Lisfranc's Clinique Chirurgicale, 8vo. Paris, 1842, from p. 182 to p. 256, are some remarks, with illustrative cases, on errors of diagnosis in uterine disease, which, though not free from the characteristic faults of that writer, will yet well repay an attentive perusal.

those positive additions to our information which we owe exclusively to the use of that instrument. The former have been very great indeed, and I think candor compels us to acknowledge that they have been due almost exclusively to persons who, not content with our previous means of investigating uterine disease, have labored to increase them by employment of instruments. The latter have certainly been less considerable, but nevertheless the speculum enables us in many instances to decide at once, and with certainty, upon the nature of a case, which otherwise we should have understood only after long and careful watching, to discover some minute polypus which the fingers alone would not have detected, to determine the source of a profuse leucorrhœal discharge, and to decide whether it is furnished by the cavity of the womb; or by the walls of the vagina; or, from the redness, congestion, or abrasion of the os uteri to infer the state of the womb generally, and thus to conduct our treatment upon the sure ground of positive observation, not upon bare presumptions. At the same time, however, that I hold the speculum to be in many cases of most essential service, I think that the endeavor of all of us should be to ascertain the minimum of frequency with which its employment is necessary. This is to be done not by decrying the instrument, still less by attributing dishonest motives to those who use it, but by soberly and honestly trying to test the value of the information which we derive from it, and learning to discriminate between those appearances which the speculum discloses that are of moment, and such as are of no importance."

Of the remaining lectures, comprised in the first part, three are devoted to menstruation and its disorders, and the rest, sixteen in number, to the uterus,—from simple inflammation to cancer,—its misplacements—including the allied misplacements of the vagina, rectum and bladder,—its inversion, and its tumors and outgrowths, this last, a most interesting section, embracing five lectures.

In never-flagging interest, in thoroughness and perspicuity, we know of no superior to Dr. West in medical authorship, without, indeed, it be that *facile princeps* of writers,—Thomas Watson, whom he, also, resembles very nearly, in a kindly appreciation of others' labors and feeling, in modesty, moderation and manliness. He is already a favorite with the profession, and this volume, for which Messrs. Blanchard and Lea deserve a generous patronage, will win the esteem and admiration of the well-built professional mind.

